# COVID-19 in Patients With Hematologic-Oncologic Risk Factors: Complications in Three Patients

**DOI:** 10.7759/cureus.12064

**Published:** 2020-12-13

**Authors:** Dawood Tafti, Matthew Kluckman, Michael C Dearborn, John Hunninghake, Sara Clayton

**Affiliations:** 1 Radiology, Brooke Army Medical Center, Fort Sam Houston, USA; 2 Critical Care Medicine, Brooke Army Medical Center, Fort Sam Houston, USA

**Keywords:** covid-19, sickle cell trait, oncology

## Abstract

The novel COVID-19 infection has demonstrated a spectrum of complications involving vascular, inflammatory, infectious, and metabolic conditions. These complications range from mild loss of smell to more severe acute respiratory distress syndrome (ARDS). Patients with more severe complications often require sedation and mechanical ventilation. Growing research has revealed the role of active malignancy and disease-in-remission status as possible risk factors contributing to the morbidity and mortality in COVID-19 patients. In our descriptive case series, we present three unique cases of complicated COVID-19 infection in patients with hematologic-oncologic risk factors and review the imaging features of their complications. The first patient was a 33-year-old male with sickle cell trait who developed rhabdomyolysis and myonecrosis of the paraspinal muscle in the setting of a physical fitness test; he subsequently developed an abscess at this site, presumably exacerbated by the hypoxemic state of his COVID-19 pneumonia. Our second patient was a 37-year-old male with COVID-19 pneumonia and a history of stage IV Non-Hodgkin's lymphoma in remission who developed spontaneous pneumomediastinum in the absence of positive pressure ventilation. The third COVID-positive patient was a 54-year-old male with a past medical history significant for grade 1 follicular non-Hodgkin’s lymphoma in remission with sputum culture positive for mycobacterium avium complex and bronchoscopy positive for candida growth. 18-FDG/PET imaging was performed and demonstrated diffuse intense uptake throughout the lungs reflecting both the COVID-19 pneumonia and the multimicrobial superinfection.

## Introduction

The severe acute respiratory syndrome coronavirus 2 (SARS-CoV-2) belongs to an enveloped, non-segmented RNA virus in the beta-coronaviridae family. Also known by its clinical infection name of COVID-19, it has resulted in significant morbidity and mortality primarily in patients with comorbidities. Described complications include super-infection from viral, bacterial, fungal etiologies, myocarditis, encephalopathy, as well as liver and kidney injury. Recent studies have investigated outcomes in COVID-19 patients with active hematologic malignancy, with evidence of worse outcomes in this cohort [1]. Parameters such as older age, immune and disease status, and performance status have been shown to be related to mortality in patients with active disease as well as those in remission [2,3]. The potential role of sickle cell disease (SCD), an immunocompromised state, in increased morbidity and mortality has also been described in various case series and studies [4,5]. One large-scale US study among 178 patients with SCD demonstrated an increased mortality rate of 7% compared to non-COVID patients with SCD [6]. Other hemoglobinopathies have also been implicated as the etiology for increased morbidity for COVID patients. The exact role of sickle cell trait as a risk factor is unclear. One recent review article suggested that COVID-19 pneumonia associated with increased oxygen demands can trigger sickle-related complications in patients with sickle cell trait [7]. 

## Case presentation

Case 1

**Figure 1:**
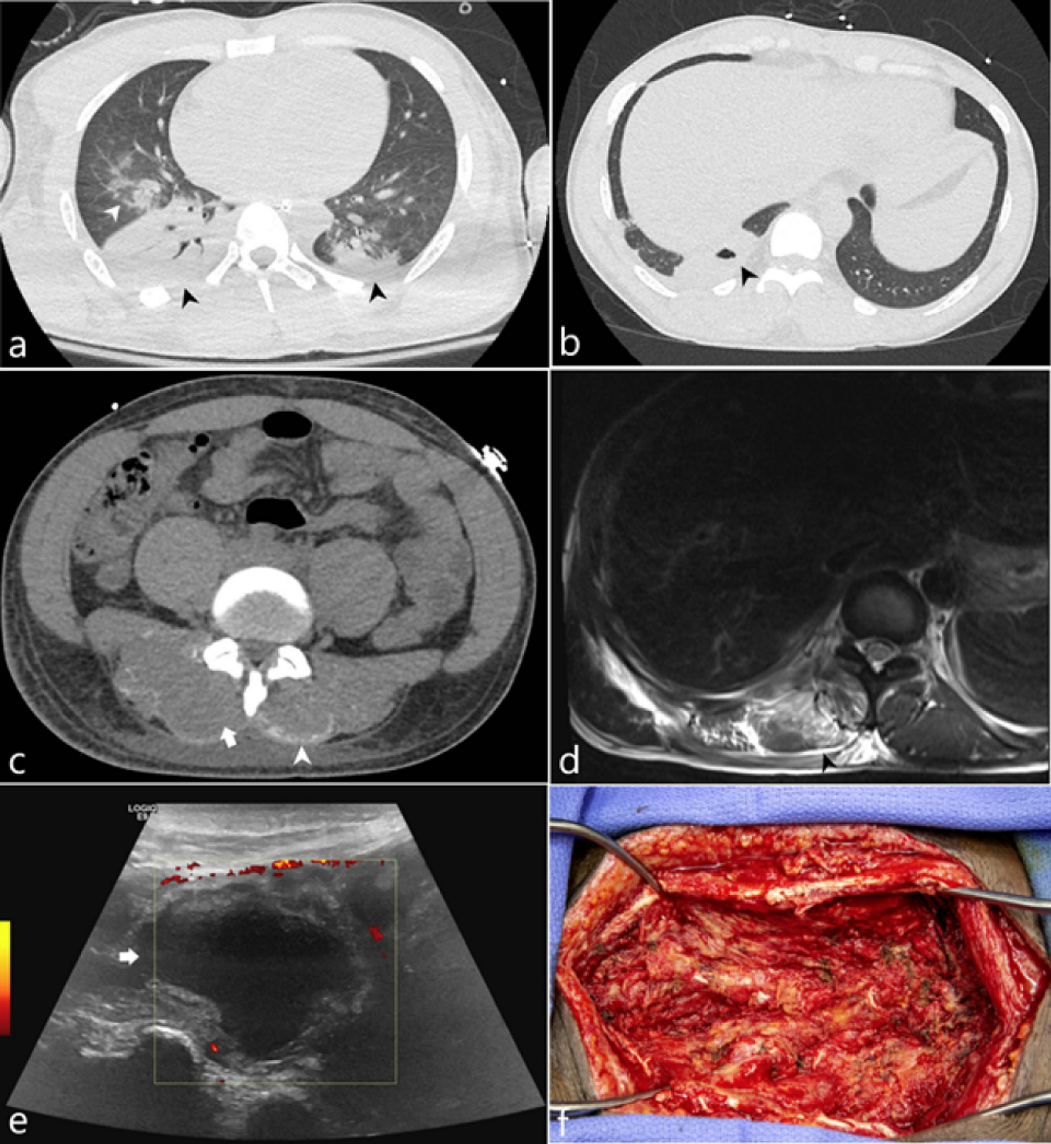
33-year-old soldier with sickle cell trait was admitted due to rhabdomyolysis complicated by COVID-19 positivity (a) Axial CT image of the lung bases acquired on hospital day 3 demonstrates consolidation (black arrowheads) and ground glass opacities (white arrowhead). (b) Axial CT image of the lung base acquired on hospital day 16 demonstrates a cavitation (black arrowhead). (c) Axial CT image of the lumbar spine at the level of the L1 vertebral body on hospital day 16 demonstrates bilateral curvilinear regions of hyperattenuation along the posterior margin of the paraspinal muscles (white arrowhead) as well as a hypoattenuating appearance of the paraspinal muscles. (d) MRI of the lower thoracic spine at the T11 level demonstrates T2 hyperintense signal (black arrowhead) of the paraspinal muscles representing edema. (e) Sonographic evaluation of the paraspinal muscle approximately 43 days from initial admission demonstrates an anechoic fluid collection (white arrow) with a thickened peripheral rim. (f) Operative image of the paraspinal soft tissues status post evacuation.

A 33-year-old male soldier with sickle cell trait was admitted due to rhabdomyolysis complicated by COVID-19 positivity. The patient developed rhabdomyolysis after collapsing on a run, and was noted to have a creatine kinase of greater than 100,000 U/L on admission. This presentation was attributed to possible exercise-collapse associated with sickle cell trait (ECAST) complicated by PCR confirmed COVID-19 positivity. His hospital course was complicated by acute renal failure requiring hemodialysis, methicillin-sensitive staph aureus pneumonia, and a pulmonary abscess. During his hospital course, the patient developed back pain and was noted to have hemorrhage into his lumbosacral musculature secondary to rhabdomyolysis induced myonecrosis (Figure 1c, d). The patient was discharged once clinically stable. Patient presented approximately three weeks later for a chief complaint of fever. Further imaging to include MRI and ultrasound of the low back demonstrated interval enlargement of the right paraspinal musculature. Fluid sampling demonstrated beta-lactamase Prevotella melaninogenica growth concerning for abscess development. The patient then underwent surgical evacuation without complication (Figure 1f).

Case 2

**Figure 2:**
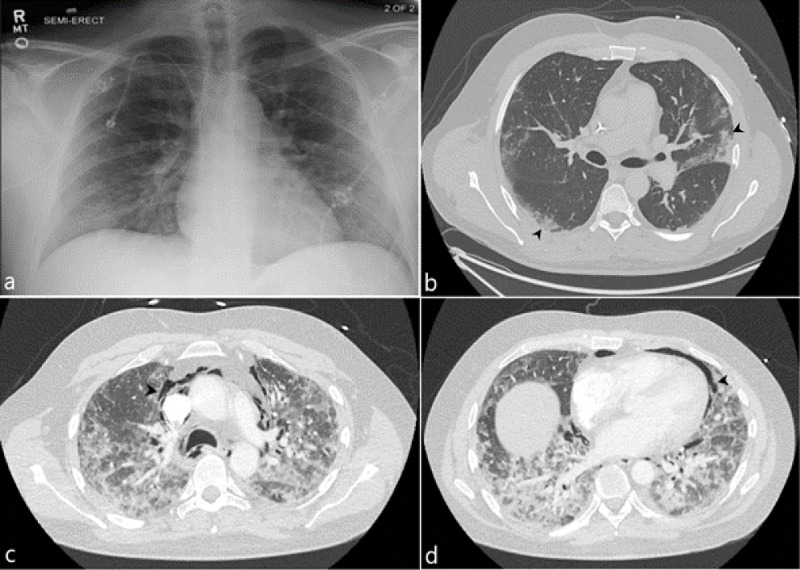
37-year-old male with spontaneous pneumomediastinum (a) Initial admission frontal chest radiograph demonstrates no significant cardiopulmonary findings. (b) Initial axial CT image of the chest demonstrates peripheral ground glass opacities (black arrowhead). (c,d) Follow up axial CT images of the chest demonstrate lucencies within the mediastinum corresponding to a new spontaneous pneumomediastinum (representative arrowheads).

A 37-year-old male with a past medical history significant for stage 4 Non-Hodgkin's lymphoma in remission and hemophagocytic lymphohistiocytosis (HLH) was admitted for COVID pneumonia in the setting of neutropenic fever. The patient had a total of four admissions during which he was managed with various antimicrobial, antiviral, and antifungal pharmacotherapy given his neutropenic state. His third admission was complicated by shortness of breath secondary to segmental and subsegmental pulmonary emboli, which were treated with a heparin drip while transitioning to apixaban. By his fourth admission, the patient presented to the emergency room for a one-day history of chest pain that radiated to his neck. The patient endorsed difficulty with swallowing and subjective changes to his voice. He also complained of shortness of breath and diffuse arthralgias. A CT neck and chest was performed which demonstrated pneumomediastinum without evidence of pneumothorax (Figure 2). The patient’s spontaneous pneumomediastinum was managed conservatively during this admission as it was clinically determined to be related to a pulmonary etiology rather than secondary to esophageal perforation.

Case 3

**Figure 3:**
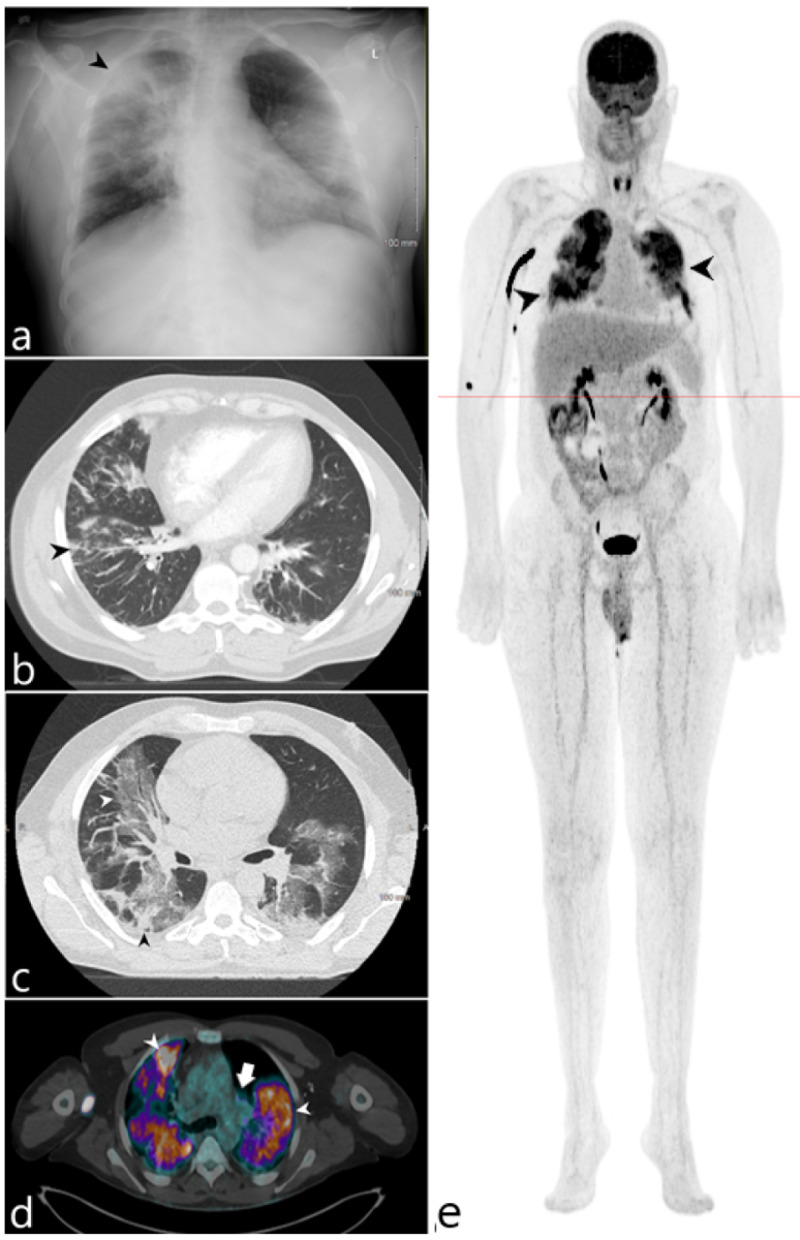
54-year-old male admitted for respiratory failure in the setting of COVID-19 pneumonia (a) Initial admission radiograph demonstrates diffuse opacities with sites of peripheral consolidation (black arrowhead). (b) Axial CT of the lower thorax performed on hospital day 10 demonstrates multifocal peribronchovascular ground glass opacity, prominently within the right lung. (c) Axial CT of the chest acquired on hospital day 22 demonstrates worsening ground glass (white arrowhead) and consolidative opacities (black arrowhead) within both lungs. (d) Fluorine-18 FDG/PET-CT axial fused series demonstrates diffuse intense radiotracer uptake within the lungs (white arrowheads) as well as within the hilar lymph nodes (white arrow). (e) 3D maximum intensity projection image similarly demonstrates intense radiotracer uptake within the lungs (black arrowheads) without additional sites of extrathoracic radiotracer uptake.

A 54-year-old male with a past medical history significant for hepatitis B, cirrhosis, psoriasis, and grade 1 follicular non-Hodgkin’s lymphoma in remission who was admitted multiple times for respiratory failure in the setting of COVID-19 pneumonia. The patient’s third admission was complicated by acute hypoxic respiratory failure, intubation, and acute kidney injury. Extensive lab work was negative for additional superinfection, including histoplasmosis, cryptococcus, coccidioides, aspergillus, and CMV. Initial bronchoscopy resulted in unsatisfactory samples for evaluation and initial blood cultures were negative. Due to the patient’s multiple admissions, oncologic history, and extended hospital course without improvement, F-18 FDG/PET study was performed to evaluate for occult infection. This revealed intense radiotracer uptake within the lungs as well as within mediastinal and hilar lymph nodes (Figure 3). There were no additional sites of extrathoracic uptake. Subsequent sputum culture was positive for mycobactrium avium complex and the patient was started on ethambutol, azithromycin and rifampin. Blood culture was positive for candida albicans and a second attempted bronchoscopy with adequate sample demonstrated candidal growth.

## Discussion

The SARS-CoV-2 represents the leading cause of morbidity and mortality in patients infected with COVID-19. Patients with SARS-CoV-2 often experience a variety of pulmonary and extrapulmonary complications and often require intubation and supportive care. Various studies have demonstrated worse outcomes in COVID-19 patients with active hematologic malignancy as well as those in remission. Hospitalized patients with hematologic malignancy, such as lymphoma, had a more severe course and a higher fatality rate [8]. These complications were most often the result of bacterial coinfections which were attributed to impaired immune system function from their disease process as well as disease treatment. Risk factors in this patient population appear to be linked to older age as well as immune and disease status [2,3].

Sickle cell trait represents the carrier state of an autosomal recessive genetic disorder. As a heterozygous state, sickle cell trait patients have one copy of mutant Hb (HbS) and one copy of a wild type allele Hb (HbA). This mutation results from a missense mutation where glutamic acid is replaced by valine in the 6th codon of the β-globulin chain. The presence of a normal allele in this recessive condition prevents the formation of polymers, and the vast majority of patients are asymptomatic. Increased oxygen demand, however, has been shown to trigger various complications in patients with sickle cell trait [9]. The role of sickle cell trait in COVID-19 associated morbidity and mortality is unclear. No literature to date has demonstrated the potential role of sickle cell trait in complications related to COVID-19 pneumonia. Although the increased mortality rates of COVID-19 in the African-American community has been attributed to socio-economic determinants of health, the role of genetic and environmental factors such as sickle cell trait has also been suggested as a possible contributor [7]. Hypoxia-triggered conditions have occurred in patients with sickle cell trait, such as exertional rhabdomyolysis, thromboembolism, and splenic infarction [10,11]. This suggests that sickle cell trait can pose as a risk factor for conditions that also result in increased oxygen demand, namely COVID-19 associated pneumonia. Our first case of myonecrosis followed by abscess formation is a good example of this potential complication in patients with sickle cell trait and rhabdomyolysis which is exacerbated by COVID-19 pneumonia.

Our second patient demonstrated a case of spontaneous pneumomediastinum, another unusual complication of COVID-19. There has been a growing number of case reports of spontaneous pneumomediastinum associated with COVID-19 pneumonia with the exact mechanism not well understood [12]. Increased alveolar pressure and diffuse alveolar injury in severe COVID-19 pneumonia is common and may make alveoli more prone to rupturing during the increased airway pressures of coughing [12]. Many cases of spontaneous pneumomediastinum in the setting of non-mechanical ventilation have also been complicated by pneumothorax [13,14]. In intubated patients with COVID-19, pneumomediastinum is likely secondary to regional barotrauma related to positive airway pressure [13,14]. This is a significant issue because patients with COVID-19 associated pneumothorax and pneumomediastinum are at a higher risk for mortality, and the magnitude of this effect seems to be even higher than that seen with prior SARS and MERS infections [15]. Due to this association, the use of a lower positive end-expiratory pressure (PEEP) and/or extracorporeal membrane oxygenation therapy has been suggested [15].

COVID-19 cases with 18F-FDG PET imaging have been a source of growing interest especially in patients with active malignancy. One of the earliest descriptions of 18F-FDG PET/CT findings in COVID-19 demonstrated a mass-like accumulation of FDG with lymphadenopathy [16]. An early case series of four COVID-19 positive patients also demonstrated FDG uptake in regions of peripheral ground-glass opacity and pulmonary consolidations in more than two pulmonary lobes as well as lymph node uptake in three of the four patients [17]. Additional case reports have gone on to establish this pattern of FDG uptake within peripheral ground glass opacities and mediastinal lymph nodes as characteristic of COVID-19 infection, even in asymptomatic individuals. These case studies also demonstrated uptake limited to the thorax and without evidence of extrathoracic uptake confirming the known pulmonary tropism of this disease. In contradistinction to the established pattern of COVID-19 infection in PET/CT, our case demonstrates diffuse florid uptake throughout the lungs, out of proportion to the ground glass opacities seen on anatomic imaging. This disparate pattern could suggest a non-COVID-19 superinfection - as was confirmed in our case by the isolation of mycobacterium avium complex and candida albicans.

Unfortunately, the use of PET/CT in the setting of COVID-19 has been limited due to the length of time 18F-FDG PET/CT procedures take to perform and the associated risks of prolonged scan time and of spreading infection in the nuclear medicine department. The implementation of aggressive disinfecting protocols and expedited PET examinations could be considered to enable PET workups in COVID-19 patients [18]. The advent of digital detector PET imaging in particular has the potential to improve access to this technology among critically ill patients who cannot be removed from the intensive care unit for prolonged periods. The improved time resolution of solid-state detectors produces a more accurate coincidence detection and therefore improves the system’s sensitivity, localization, and counting efficiency. This has the potential to reduce scan times of such examinations by up to 40% and may make PET examinations more feasible in the critically ill population.

In addition to the utility of PET/CT patterns in evaluating known COVID-19 patients, it is important to note that many case reports have demonstrated the incidental detection of COVID-19 pneumonia in asymptomatic carriers both with and without malignancy. In fact, between 40% and 45% of patients with COVID-19 may be asymptomatic [19]. These patients may play a significant role in the ongoing pandemic as they may transmit the virus via the silent spread. It is therefore imperative for us as radiologists to be aware of the typical pattern of COVID-19 disease on PET imaging so that we can direct the patient toward timely diagnostic testing and isolation.

## Conclusions

Growing research into the novel COVID-19 epidemic has shown a wide range of pulmonary and extrapulmonary complications secondary to this viral illness. Various risk factors, including active and prior malignancy, have demonstrated their importance in predicting outcomes for COVID-19 patients. Additional associations of COVID-19 with pneumothorax and pneumomediastinum are important considerations and have been suggested to have prognostic significance. The exact role of 18F-FDG PET/CT in the evaluation of patients infected with COVID-19 is evolving and may have a limited utility in certain cases. While increased morbidity and mortality in patients with sickle cell disease has been well established, more research into the role of morbidity associated with sickle cell trait is warranted, especially in light of the severe complications suffered by the first patient in our series.
